# Exploring the Present and Imagining the Future Landscape of Onconephrology

**DOI:** 10.34067/KID.0000000000000544

**Published:** 2024-08-26

**Authors:** Abinet M. Aklilu, Anushree C. Shirali

**Affiliations:** 1 Section of Nephrology, Department of Internal Medicine, Yale University School of Medicine, New Haven, Connecticut; 2 Clinical and Translational Research Accelerator, Yale University, New Haven, Connecticut

**Keywords:** onco-nephrology

From its beginnings in 2010 as an area of interest among nephrologists, the field of onconephrology has now expanded to become a subspecialty within nephrology. The inevitable creation and widespread adoption of this field has paralleled rapid advancements in both the diagnosis and treatment of cancer. Specifically, as more precise and effective anticancer therapies were developed for clinical use, a specialized field bridging the world of oncology and nephrology became necessary for multiple reasons.^1^ Key among these reasons were the need for recognition and understanding of (*1*) off-target acute and chronic effects of established and novel anticancer therapeutics on kidney function; (*2*) pharmacokinetics of anticancer drugs cleared or metabolized by the kidney, particularly in patients with kidney injury or disease; (*3*) direct acute effects of cancer on kidney function; and (*4*) longitudinal care of kidney disease in long-term survivors of cancer. Over a decade later, the field continues to advance with even more reasons for its expansion.

The growth of onconephrology over the past 10–15 years is as impressive as the practice advancements in oncology. During this time, the field has witnessed, among many achievements, the formation of training programs at major cancer centers, the founding of its own flagship journal, the publication of curricula through the American Society of Nephrology and *American Journal of Kidney Diseases*,^2,3^ the inclusion of dedicated sessions at national and international nephrology conferences, and the development of national and international societies. Alongside this, a growing number of nephrology trainees are choosing to focus on the care of kidney disease in patients with cancer, leading many hospitals to establish dedicated onconephrology clinics. Furthermore, there has been an exponential growth in publications related to onconephrology, ranging from review and perspective articles to original research. Some of these latter have characterized the incidence, patterns, and prognosis of kidney injury associated with various traditional chemotherapeutics, immunotherapy, and targeted therapies, as well as hematopoietic stem cell transplant. There are ongoing efforts to investigate and develop noninvasive diagnostic tools for early diagnosis of AKI in patients receiving immunotherapy^4^ and for the development of risk prediction algorithms^5^ to improve patient care and outcomes.

With these notable seminal events in the field, we are pleased to introduce a new series on onconephrology in *Kidney360*. The series kicks off with an expert perspective on specialized training in onconephrology and will contain nine additional articles in the following areas: GFR assessment in patients with cancer and kidney disease; novel biomarkers and imaging for AKI diagnosis in patients with cancer; treatment of AKI associated with high-dose methotrexate; treatment of AKI associated with small molecules and targeted therapies; treatment of AKI associated with immunotherapy; treatment of kidney disease in systemic amyloidosis, transplant onconephrology; and palliative care and ethical considerations in managing kidney disease in patients with advanced malignancies. In accordance with the mission of *Kidney360*, these articles are collective efforts of clinician experts across different continents to foster international collaboration and to ensure inclusion of diverse perspectives. Furthermore, to ensure a global reach without a prohibitive paywall, these articles will be released successively over the next 6 months on the open-access *Kidney360* website.

This series will highlight topics in onconephrology that most nephrologists are likely to encounter in their practice. The aim is not only educational but also to highlight gaps in our understanding of the myriad of ways that cancer and its treatment affect kidney function. As such, parallel to the aim of clinical education, the hope is that this series will also stimulate research ideas. Onconephrology is a nascent field, and there are many potential areas of inquiry that further highlight the need not only for clinical onconephrology but also robust research programs that examine the intersection of cancer and the kidney.

Among topics that need attention is the care of long-term survivors of cancer. Every year, approximately 19 million individuals are diagnosed with cancer globally, of which two million live in the United States. According to the National Cancer Institute's Surveillance, Epidemiology, and End Results program statistics, in 2024, there are around 18 million cancer survivors in the United States with nearly 1/5 having lived over 20 years since their diagnosis. This is projected to further increase to 26 million in 15 years. CKD is more prevalent among cancer survivors compared with the general population with an estimated up to 25% of patients with cancer having an estimated GFR below 60 ml/min.^6^ CKD or rapid GFR decline may be even more common in survivors of some cancers and in individuals treated with hematopoietic stem cell transplant or immunotherapy.^7^ As cancer survivorship improves, the population with cancer-associated CKD is expected to grow. Improved cancer survivorship, particularly in children, should lead to a concerted effort in improving kidney protective strategies during treatment and the long-term care of this growing population.

There are additional settings in patient care that require the specialized input of those engaged in the field of onconephrology. This includes the identification of nephrotoxic dysproteinemias that otherwise do not meet hematologic criteria for treatment. The recognition of this entity, now known as monoclonal gammopathies of renal significance, as a disease requiring prompt diagnosis and treatment^8^ should improve diagnosis and outcomes for this population, provided there is prompt referral to nephrologists experienced in the care of such patients. Furthermore, therapeutic advancement has lately brought attention to areas of complex clinical decision making, such as immunotherapy in transplant recipients and risk of allograft rejection,^9^ advanced kidney disease in the setting of advanced malignancy, and cancer screening in advanced kidney disease.^10^

While acknowledging the field's current progress in the care of patients with cancer and kidney disease, it is also worth recognizing areas where there is need and potential for involvement and growth. Cancer and kidney disease are global diseases, but there has been great disparity in equitable access to cancer care and to kidney care.^11,12^ With ongoing efforts to improve cancer detection at national and regional levels worldwide, the incidence of cancer is increasing in low- and middle-income countries^11^ where the overwhelming majority of cancer and kidney-related deaths occur. At this time, in addition to improving access to cancer treatment, there is need for nephrology and palliative care services for patients with cancer. The time is now for collaborative global efforts to improve access to these services, develop onconephrology clinical programs, establish and maintain patient registries, and promote diverse and international representation in clinical trials.

**Figure 1:**
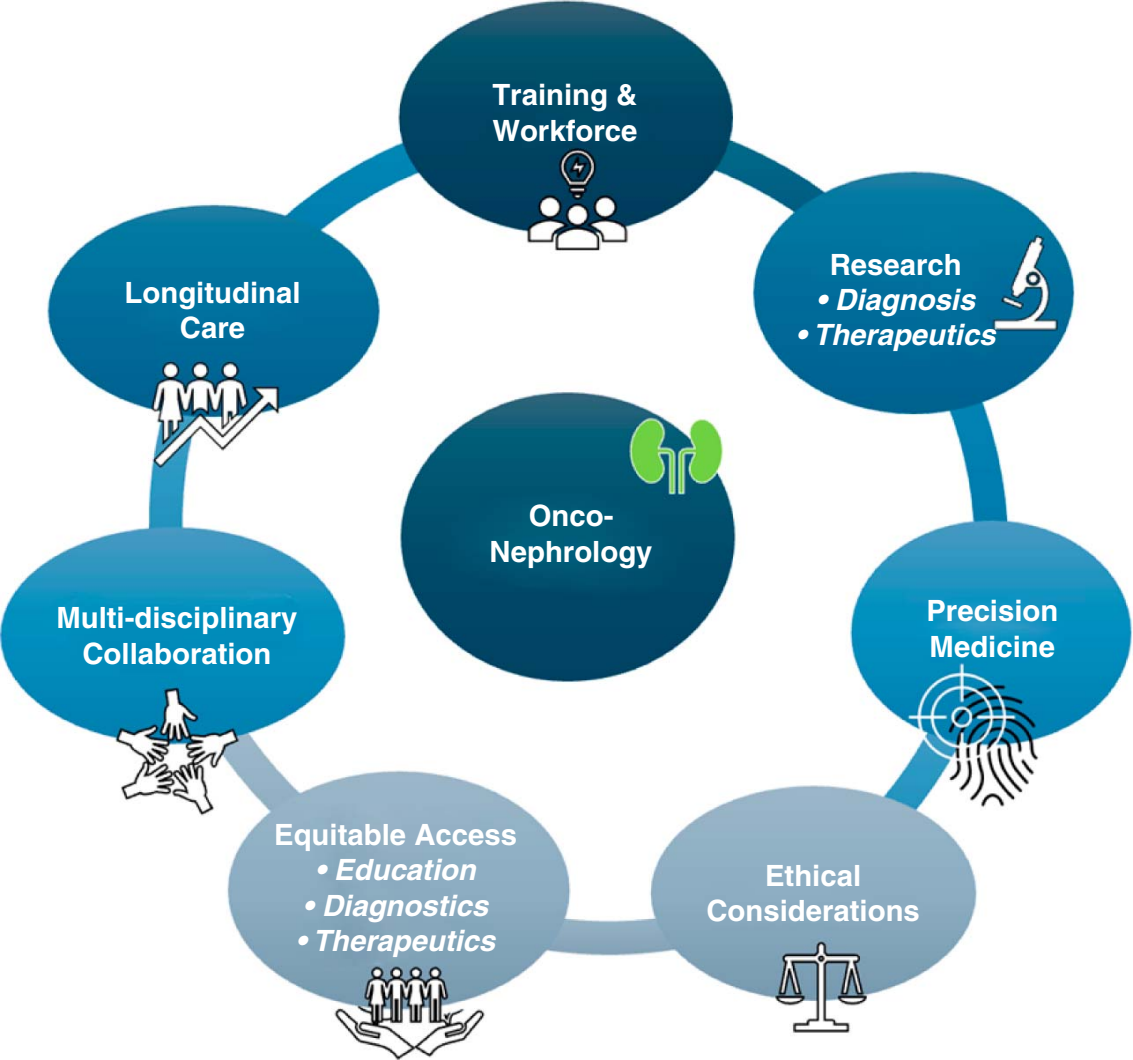
Onconephrology: a new paradigm.

There are other topics that deserve clinical and research consideration in onconephrology. For example, there is a need to improve estimation of kidney function in patients with cancer because inaccurate drug dosing can have detrimental consequences. There is also need for early and phenotype-specific biomarkers for diagnosis and monitoring of AKI, for timely identification of individuals at high risk of AKI, and for efficient clinical decision support tools to facilitate AKI management in patients with cancer. The ongoing digital revolution with the expansion and growing adoption of artificial intelligence could expedite efforts toward some of these goals. Figure 1 shows an updated perspective on the future of onconephrology. With this, we can hope for a productive future of the field that builds upon past successes.
